# Postoperative nutritional outcomes and quality of life-related complications of proximal *versus* total gastrectomy for upper-third early gastric cancer: a meta-analysis

**DOI:** 10.1038/s41598-020-78458-0

**Published:** 2020-12-08

**Authors:** Inhyeok Lee, Youjin Oh, Shin- Hoo Park, Yeongkeun Kwon, Sungsoo Park

**Affiliations:** 1 Department of Medicine, Korea University College of Medicine, Seoul, Republic of Korea; 2 Division of Foregut Surgery, Korea University College of Medicine, Goryeodae-ro 73, Seongbuk-gu, Seoul, 02841 Republic of Korea

**Keywords:** Gastroenterology, Medical research, Oncology

## Abstract

Although proximal gastrectomy (PG) provides superior nutritional outcomes over total gastrectomy (TG) in upper-third early gastric cancer (EGC), surgeons are reluctant to perform PG due to the high rate of postoperative reflux. This meta-analysis aimed to comprehensively compare operative outcomes, nutritional outcomes, and quality of life-related complications between TG and PG performed with esophagogastrostomy (EG), jejunal interposition, or double-tract reconstruction (DTR) to reduce reflux after PG. After searching PubMed, Embase, Medline, and Web of Science databases, 25 studies comparing PG with TG in upper-third EGC published up to October 2020 were identified. PG with DTR was similar to TG regarding operative outcomes. Patients who underwent PG with DTR had less weight reduction (weighted mean difference [WMD] 4.29; 95% confidence interval [0.51–8.07]), reduced hemoglobin loss (WMD 5.74; [2.56–8.93]), and reduced vitamin B_12_ supplementation requirement (odds ratio [OR] 0.06; [0.00–0.89]) compared to patients who underwent TG. PG with EG caused more reflux (OR 5.18; [2.03–13.24]) and anastomotic stenosis (OR 3.94; [2.40–6.46]) than TG. However, PG with DTR was similar to TG regarding quality of life-related complications including reflux, anastomotic stenosis, and leakage. Hence, PG with DTR can be recommended for patients with upper-third EGC considering its superior postoperative nutritional outcomes.

## Introduction

Nationwide surveys from East Asia have reported that the number of early gastric cancer (EGC) and upper-third gastric cancer cases has increased^1–3^, despite the decreasing incidence of gastric cancer globally^4,5^. According to the treatment guidelines by Korean and Japanese Gastric Cancer Associations, proximal gastrectomy (PG) and total gastrectomy (TG) can be considered an effective curative treatment for clinical stage IA cancer in the upper-third of the stomach^6,7^. However, in Korea, which has the highest incidence of gastric cancer worldwide, PG accounted for only 1.1% of all gastric cancer surgeries, while upper-third gastric cancer accounted for 16.0% of all gastric cancer cases in 2014^1^.

TG is known to cause nutritional disadvantages compared to PG, especially in vitamin B_12_ because of the decrease in the intrinsic factor secreted by parietal cells located in the distal stomach^8^. Moreover, lesser post-gastrectomy syndromes occur after PG because of the retained storage capacity of the distal stomach and preserved function of the pylorus^9^. Despite the advantages in preserving the distal stomach, surgeons are reluctant to perform PG with esophagogastrostomy (EG) because of the notorious gastroesophageal reflux that occurs after PG^10^.

Several novel types of anastomosis to reduce gastroesophageal reflux have been created and tested including jejunal interposition (JI), jejunal pouch interposition (JPI), and double-tract reconstruction (DTR)^11–13^. Although DTR has gained popularity due to its easier laparoscopic approach than other methods, there is insufficient evidence regarding patient outcomes. Additionally, the choice of the method for anastomosis depends on the surgeons’ preference due to the unavailability of standardized guidelines^14^. To help surgeons decide regarding optimal anastomosis for patients with EGC undergoing PG, a comprehensive analysis of PG with several anastomotic methods is needed.

This systematic review and meta-analysis aimed to comprehensively compare TG and PG with several anastomotic methods to help surgeons choose the optimal anastomotic method and improve clinical outcomes in patients undergoing surgery. To achieve this goal, we analyzed operative outcomes, nutritional outcomes, and quality of life-related complications in patients with upper-third EGC who underwent either TG or PG, especially considering the various anastomotic methods.

## Methods

### Literature search strategy

This meta-analysis was performed according to the Preferred Reporting Items for Systematic Reviews and Meta-Analyses (PRISMA) guidelines to improve the quality of the systematic review^15^. A comprehensive literature search was performed using PubMed, Embase, Medline, and Web of Science databases. Related articles and the bibliography of the identified articles were also reviewed to prevent overlooking articles that may not have been indexed. Articles with suitable data published in English were included. Furthermore, articles published up to October 2020 were explored.

The search terms used were “early gastric cancer”, “proximal gastrectomy”, and “total gastrectomy” with the Medical Subject Headings (MeSH) terms, “Stomach Neoplasm” and “Gastrectomy”. Abstracts and full texts of all articles were screened and reviewed by two authors (I.H.L. and Y.J.O.) based on consensus agreement. The search was conducted up to October 22, 2020.

### Study selection criteria

Studies that met the following criteria were included: (1) studies comparing PG and TG; (2) studies presenting the criteria for EGC including the stage or depth of invasion; and (3) studies including operative outcomes, nutritional outcomes, or quality of life-related complication. In cases where two studies were conducted using identical cohorts, the study with more comprehensive nutritional or operative outcomes was selected. The study by Kosuga et al. was selected over the study by Ichikawa et al. because it had more assessable data^16,17^.

Studies were excluded based on the following criteria: (1) lack of necessary statistical data such as variance; (2) non-English publications; (3) posters, review papers, comments, and abstract-only papers; (4) lack of criteria defining EGC according to stage and depth of invasion; and (5) the use of heterogeneous operation type. The studies by Kano et al*.* and Hayami et al*.*, which compared TG and PG with EG combined with DFT, were excluded due to insufficient data and the heterogeneity in operative outcomes and gastroesophageal reflux due to the additional anti-reflux procedure of DFT^18,19^.

### Data extraction and bias assessment

Data extraction from the included studies was performed by two authors independently (I.H.L. and Y.J.O.). The extracted data included (1) background of the study (author names, year of publication, study design, hospital location, nationality, and number of patients in each arm); (2) cohort characteristics (anastomosis type, age, sex, body mass index (BMI), and tumor size); (3) operative outcomes (operation time, intraoperative blood loss, postoperative complications, and postoperative hospital stay); (4) postoperative nutritional outcomes (weight change, hemoglobin change, postoperative vitamin B_12_ supplementation requirement, postoperative iron supplementation requirement, serum albumin change, and lymphocyte count change); and (5) postoperative quality of life-related complications (gastroesophageal reflux, anastomotic stenosis, and anastomotic leakage). Twenty-five non-randomized studies were assessed using the Risk of Bias In Non-Randomized Studies—of Interventions (ROBINS-I) tool, which is a useful tool for evaluating non-randomized studies^20^. Two studies were found to have serious bias due to bias in the measurement of outcomes and in the selection of the reported result (Table 1). Funnel plots were used to evaluate publication bias; and no bias was detected (see Supplementary Fig. S1 online).

**Table 1:** Quality assessment for 25 non-randomized control trial studies using the Risk of Bias In Non-Randomised Studies—of Interventions (ROBINS-I) tool.

	Confounders	Selection of patients	Classification of interventions	Deviations from intended interventions	Missing data	Measurement of outcome	Selection of the reported result	Overall bias
**1. PG with DTR**								
Ko et al. (2019)^32^	Low	Low	Low	No information	Moderate	Low	Low	Moderate
Nomura et al. (2019)^22^	Moderate	Low	Moderate	No information	Low	Low	Low	Moderate
Cho et al. (2018)^33^	Low	Moderate	Low	No information	Moderate	Low	Low	Moderate
Furukawa et al. (2018)^21^	Low	Low	Low	No information	Moderate	Low	Low	Moderate
Park et al. (2018)^34^	Moderate	Low	Low	No information	Moderate	Low	Low	Moderate
Sugiyama et al. (2018)^35^	Moderate	Low	Low	No information	Low	Low	Low	Moderate
Jung et al. (2017)^36^	Low	Moderate	Low	No information	Moderate	Low	Low	Moderate
Kim and Kim (2016)^37^	Low	Moderate	Low	No information	Moderate	Low	Low	Moderate
**2. PG with Non-DTR**								
Asaoka et al. (2019)^38^	Moderate	Moderate	Low	No information	Moderate	Low	Low	Moderate
Zhou et al. (2019)^39^	Moderate	Moderate	Low	No information	Moderate	Low	Low	Moderate
Ushimaru et al. (2018)^40^	Low	Low	Low	No information	Low	Low	Low	Low
Nishigori et al. (2017)^41^	Moderate	Low	Low	Moderate	Moderate	Low	Low	Moderate
Hosoda et al. (2016)^42^	Low	Low	Low	No information	Moderate	Low	Low	Moderate
Huh et al. (2015)^43^	Moderate	Low	Low	No information	Moderate	Low	Low	Moderate
Kosuga et al. (2015)^16^	Moderate	Low	Low	Moderate	Moderate	Low	Low	Moderate
Ohashi et al. (2015)^44^	Moderate	Low	Low	No information	Moderate	Low	Low	Moderate
Isobe et al. (2014)^23^	Moderate	Low	Low	No information	Moderate	Low	Low	Moderate
Masuzawa et al. (2014)^45^	Moderate	Low	Low	No information	Moderate	Low	Low	Moderate
Son et al. (2014)^46^	Moderate	Low	Low	No information	No information	Low	Serious	Serious
Ahn et al. (2013)^47^	Moderate	Low	Low	No information	Moderate	Low	Low	Moderate
Nozaki et al. (2013)^48^	Moderate	Low	Low	No information	Moderate	Serious	Low	Serious
Namikawa et al. (2012)^49^	Low	Low	Low	No information	Low	Low	Low	Low
An et al. (2008)^50^	Moderate	Low	Low	No information	Low	Low	Low	Low
Kitano et al. (2007)^51^	Moderate	Low	Moderate	Moderate	Low	Low	Low	Moderate
Kondoh et al. (2007)^52^	Low	Low	Low	No information	Low	Low	Low	Low

### Subgroup analysis and sensitivity analysis

In this meta-analysis, the included studies were divided into subgroups according to three types of anastomosis performed in PG: PG with DTR, PG with EG, and PG with JI. The PG with JI subgroup included PG with JI and PG with JPI; subgroup analyses were conducted in terms of operative outcomes, nutritional outcomes, and postoperative quality of life-related complications.

If a study included multiple types of anastomosis in PG without classification, the study was classified into the subgroup with the most common type accounting for more than 75% of the overall cohort. The studies by Furukawa et al. and Zhou et al*.* were classified into the DTR and EG subgroups respectively because the proportion of the most common type of anastomosis in the overall cohort was > 75% in both studies (78% and 89%, respectively)^21,39^. Considering the possibility of heterogeneity and bias imposed due to the classification, sensitivity analyses excluding the studies by Furukawa et al. and Zhou et al*.* were conducted in terms of operative outcomes, postoperative outcomes, and postoperative quality of life-related complications.

If a study included comparisons between TG and PG with two different types of anastomosis, the study was classified into both subgroups. Hence, the studies by Nomura et al*.* and Isobe et al*.* were classified into PG with two different subgroups; Nomura et al*.* (DTR and JI) and Isobe et al. (EG and JI)^22,23^. While performing subgroup analyses using these studies, it was inevitable to calculate the events in the TG group repeatedly because the data of two different subgroups were exploited. To minimize the bias incurred in repeatedly calculating the events in the TG group of the studies by Nomura et al*.* and Isobe et al*.*, we performed additional analyses where the events in the TG and PG groups were counted only once by combining the data from each anastomosis type.

For operative parameters, subgroup analyses were conducted according to the type of surgery: open or laparoscopic surgery. For the postoperative complications, the onset and the severity measured according to the Clavien–Dindo (C–D) classification^24^. Subgroup analyses of gastroesophageal reflux were conducted according to the reported features (reflux symptoms or reflux esophagitis confirmed endoscopically), and their severity was measured using the Visick score or Los Angeles (LA) Classification System, respectively^25,26^.

### Statistical analysis

All analyses were conducted using the statistical software Review Manager (RevMan Version 5.3; The Nordic Cochrane Centre, Copenhagen, Denmark). The outcomes of dichotomous variables are presented as odds ratios (ORs), while the outcomes of continuous variables are presented as weighted mean differences (WMDs). All results are presented with 95% confidence intervals (CIs) using the Mantel–Haenszel method^27^. Heterogeneity was measured using Higgins I^2^ statistics and the Cochran Q test^28^. The meta-analyses were performed using a random-effects model because the cohorts of the included studies from different institutions were not identical or homogenous^29^. If the only accessible data were median and interquartile ranges, the mean and standard deviation were estimated from the median, interquartile ranges, and the size of included studies according to the references^30,31^. A p-value of less than 0.05 was considered statistically significant.

## Results

### Study characteristics

**Figure 1:**
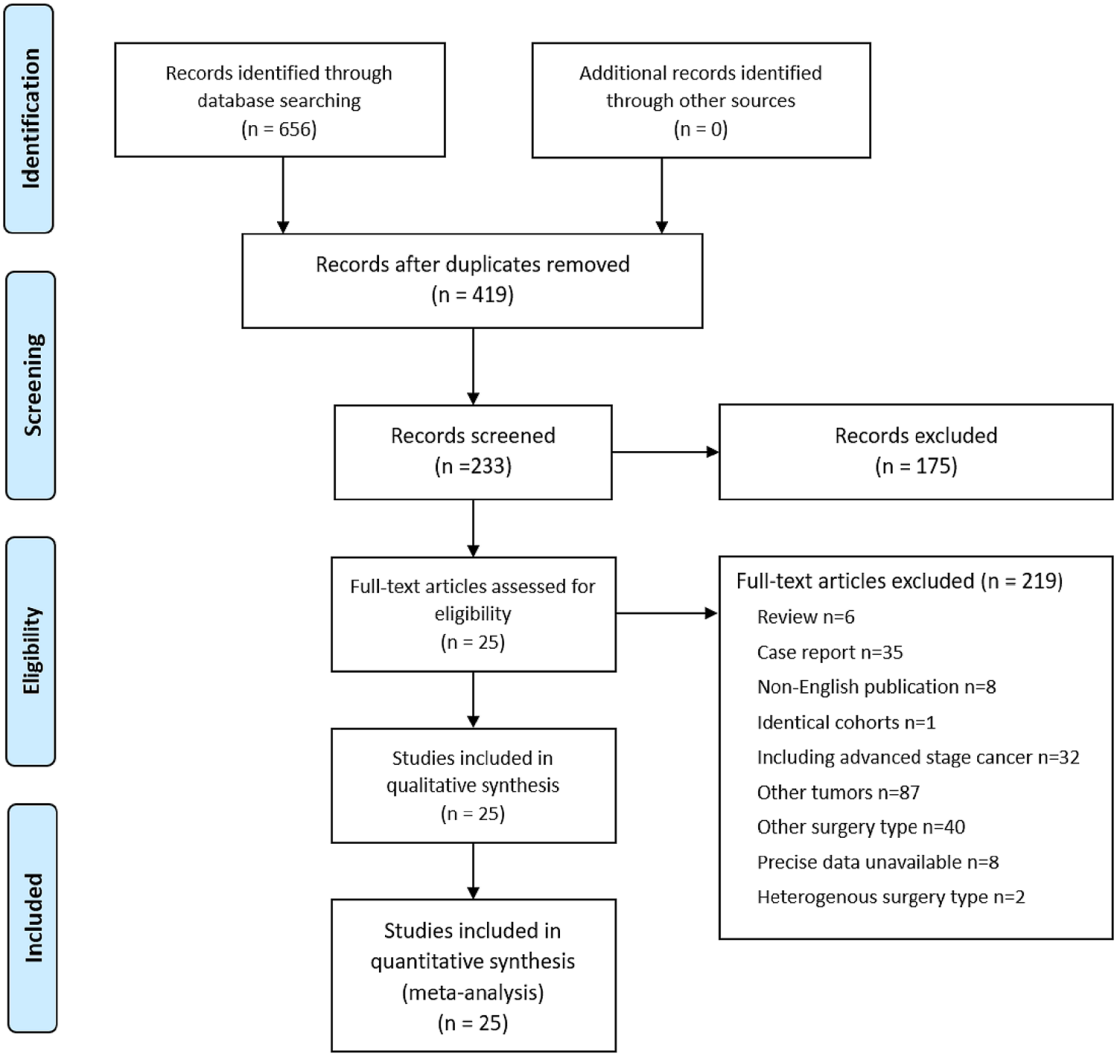
PRISMA flow diagram for the meta-analysis.

Twenty-five studies, including 3,058 patients (1,287 patients who underwent PG and 1,771 patients who underwent TG) were selected for the meta-analyses (Fig. 1 and Table 2). All studies were observational studies; 4 case-control studies and 21 retrospective cohort studies. PG with DTR was performed in 8 studies including 696 patients, while PG with non-DTR was performed in 18 studies including 2,392 patients. PG with EG was performed in 13 studies including 1,781 patients and PG with JI was performed in 6 studies including 649 patients.

**Table 2:** Summary of the studies included in this meta-analysis.

Study (publish year)	Study Design	Period of surgery	Country	Patients criteria	Approach (N)	Surgery type	Anastomosis type	Age (years)	Male (%)	BMI (kg/m^2^)
**1. PG with DTR**										
Ko et al. (2019)^32^	C	2008–2016	Korea	cT1, cT2	PG (52)	O/L	DTR	61.5 ± 12.5	67.3	23.7 ± 3.1
					TG (52)	O/L	RY	63.0 ± 9.2	67.3	23.4 ± 2.9
Nomura et al. (2019)^22^	R	2012–2016	Japan	cStage I	PG (15)	L	DTR	65.7 ± 10.6	89.7	n.s
					PG (15)	L	JI	69.3 ± 6.0	73.3	n.s
					TG (30)	L	RY	68.5 ± 8.3	70	n.s
Cho et al. (2018)^33^	R	2014–2015	Korea	pStage I	PG (38)	L/R	DTR	55.8 ± 11.6	84.2	24.2 ± 3.1
					TG (42)	L/R	RY	59.3 ± 11.8	73.8	23.5 ± 3.0
Furukawa et al. (2018)^21^	R	2010–2016	Japan	cStage I	PG (27)	L	DTR/EG	70 (59–84)†	81.5	22.8 (19.3–26.8)†
					TG (48)	L	RY	63.5 (29–82)†	72.9	22.2 (13.9–26.5)†
Park et al. (2018)^34^	R	2011–2015	Korea	cStage I	PG (34)	L	DTR	64.1 ± 12.2	76.5	23.1 ± 3.2
					TG (46)	L	RY	56.7 ± 11.8	47.8	22.9 ± 3.4
Sugiyama et al. (2018)^35^	R	2013–2016	Japan	cStage IA	PG (10)	L	DTR	65.6 ± 3.8	70	21.3 ± 1.0
					TG (20)	L	RY	68.6 ± 2.7	85	23.7 ± 0.7
Jung et al. (2017)^36^	R	2003–2015	Korea	pStage I	PG (92)	L	DTR	59.8 ± 11.4	83.7	23.5 ± 2.7
					TG (156)	L	RY	58.7 ± 10.8	76.9	23.9 ± 3.3
Kim and Kim (2016)^37^	C	2009–2014	Korea	cStage IA	PG (17)	L	DTR	64.7 ± 9.9	82.4	24.2 ± 3.8
					TG (17)	L	RY	60.9 ± 12.9	58.8	23.4 ± 5.0
**2. PG with non-DTR**										
Asaoka et al. (2019)^38^	R	2010–2014	Japan	cStage I	PG (39)	O	EG/ JI	66 (38–86)†	84.6	23.2 ± 3.10
					TG (73)	O	RY	70 (46–860)†	78.1	23.4 + ± 3.20
Zhou et al. (2019)^39^	R	1980–2012	China	pT1, pT2	PG (67)	n.s	EG/DTR/JI	n.s	n.s	n.s
					TG (47)	n.s	RY	n.s	n.s	n.s
Ushimaru et al. (2018)^40^	C	2004–2013	Japan	cStage I	PG (39)	O	EG	67 (44–83)†	82.1	23.0 (18.3–28.0)†
					TG (39)	O	RY	69 (34–83)†	79.5	22.7 (16.6–30.9)†
Nishigori et al. (2017)^41^	R	2006–2014	Japan	cStage I	PG (20)	L	EG	66.2 ± 13.4	75	23.4 ± 3.8
					TG (42)	L	RY	64.4 ± 12.2	67	22.8 ± 3.6
Hosoda et al. (2016)^42^	C	2009–2014	Japan	cT1	PG (16)	L	EG	69.2 ± 8.2	75	22.9 ± 2.6
					TG (16)	L	RY	67.7 ± 8.4	69	23.6 ± 3.7
Huh et al. (2015)^43^	R	2002–2012	Korea	cT1	PG (192)	O/L	EG	59.7 ± 11.2	67.7	24.3 ± 2.9
					TG (157)	O	RY	57.4 ± 11.9	73.0	23.5 ± 3.1
Kosuga et al. (2015)^16^	R	2009–2014	Japan	cStage I	PG (25)	L	EG	66 (41–80)†	68	22.3 (17.7–28.0)†
					TG (52)	L	RY	67 (40–89)†	86.5	23.6 (19.0–42.8)†
Ohashi et al. (2015)^44^	R	2007–2012	Japan	cT1	PG (65)	O	JI	67 (37–77)†	85	23.7 ± 2.9
					TG (117)	O	RY	67 (30–84)†	71	23.5 ± 3.4
Isobe et al. (2014)^23^	R	1989–2008	Japan	pT1	PG (66)	n.s	EG	71.6 ± 9.6	78.8	n.s
					PG (23)	n.s	JI	59.4 ± 8.5	78.3	n.s
					PG (12)	n.s	JPI	59.9 ± 9.8	75	n.s
					TG (38)	n.s	n.s	61.3 ± 9.5	78.9	n.s
Masuzawa et al. (2014)^45^	R	1998–2005	Japan	pT1	PG (49)	O/L	EG	64.0 ± 7.7	73.5	n.s
					PG (32)	O/L	JI	65.0 ± 12.1	78.1	n.s
					TG (122)	O/L	RY	63.0 ± 10.0	73.0	n.s
Son et al. (2014)^46^	R	2001–2008	Korea	cT1	PG (64)	O	EG	58.0 ± 13.3	67.2	n.s
					TG (106)	O	RY	61.3 ± 10.3	71.7	n.s
Ahn et al. (2013)^47^	R	2003–2009	Korea	cStage I	PG (50)	L	EG	58.8 ± 12.1	72	24.2 ± 3.7
					TG (81)	L	RY	59.7 ± 11.8	69.1	23.6 ± 3.4
Nozaki et al. (2013)^48^	R	1999–2008	Japan	cStage I	PG (102)	O	JI	67 (44–85)†	77	n.s
					TG (49)	O	RY	71 (34–86)†	73	n.s
Namikawa et al. (2012)^49^	R	2004–2010	Japan	cStage I	PG (22)	O	JPI	63 (49–77)†	72.7	n.s
					TG (22)	O	RY	65 (38–87)†	77.3	n.s
An et al. (2008)^50^	R	2000–2005	Korea	pT1	PG (89)	n.s	EG	56.6 ± 10.9	69.7	n.s
					TG (334)	n.s	RY	55.1 ± 12.1	68.3	n.s
Kitano et al. (2007)^51^	R	1994–2003	Japan	pT1	PG (54)	L	EG	63.7 ± 9	75.9	§
					TG (55)	L	RY	62.1 ± 12	81.8	§
Kondoh et al. (2007)^52^	R	1997–2004	Japan	pStage IA	PG (10)	O	EG	67.8 ± 5.9	90	23.6 ± 1.8
					TG (10)	O	RY	61.4 ± 8.5	90	24.1 ± 2.7

*Values presented as mean ± standard deviation unless indicated otherwise.

^†^Values with parenthesis presented as median (range).

^§^Data presented as range are shown in Supplementary Table S1 online.

BMI, body mass index; C, case–control; R, retrospective cohort; PG, proximal gastrectomy; TG, total gastrectomy; L, laparoscopic; O, open; R, robotic; DTR, double-tract reconstruction; EG, esophagogastrostomy; JI, jejunal interposition; JPI, jejunal pouch interposition; RY, Roux-en Y; n.s., not stated.

The characteristics of the studies used in the meta-analyses are described in Table 2^16,21–23,32–52^. Age, sex, BMI, and tumor size are presented as mean ± standard deviation or median (interquartile range). Further, in one study, patients were categorized into 3 subgroups according to BMI (Supplementary Table S1 online). The criteria for EGC varied between studies, and the definition of EGC was clinical or pathological T1, or stage I gastric cancer in most studies. Three studies established the definition of EGC more narrowly as clinical or pathological stage IA^35,37,52^. In contrast, two studies defined EGC more widely as clinical or as pathological T1 or T2 gastric cancer^32,39^.

### Operative outcomes

**Figure 2:**
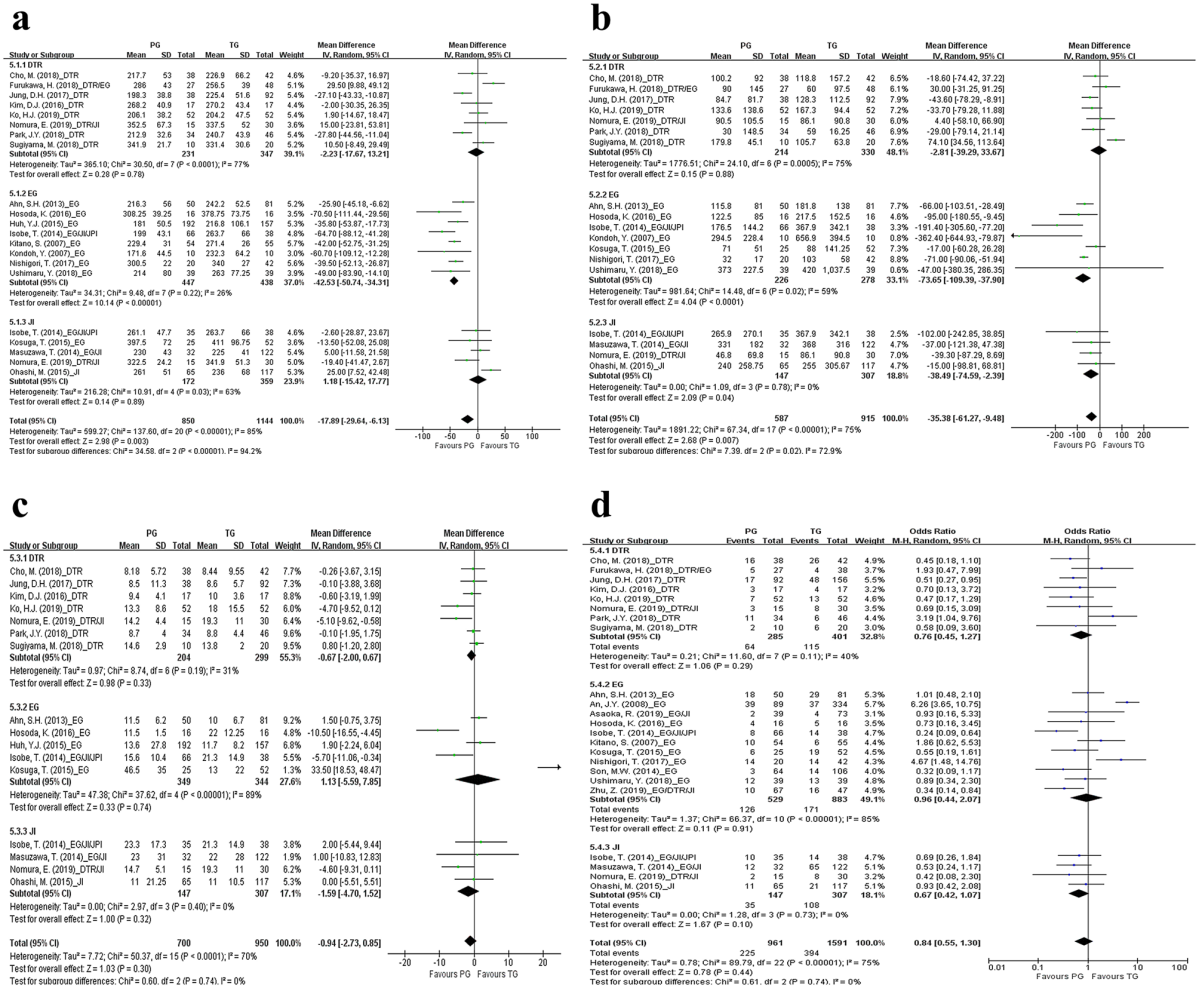
Forest plots for comparing operative outcomes between proximal gastrectomy and total gastrectomy including (**a**) operation time, (**b**) intraoperative blood loss, (**c**) postoperative hospital stay, and (**d**) postoperative complications. The meta-analyses were performed using the Mantel–Haenszel random-effect model. Weighted mean differences or odds ratios are shown with 95% confidence intervals. PG, proximal gastrectomy; TG, total gastrectomy; DTR, double-tract reconstruction; EG, esophagogastrostomy; JI, jejunal interposition.

Operative time, intraoperative blood loss, postoperative complications, and duration of postoperative hospital stay were included in the meta-analysis of operative outcomes. In the analysis of the operative outcomes, the PG group was subdivided into PG with DTR, PG with EG, and PG with JI subgroups. The operative time was shorter in PG with EG (WMD − 42.53, CI − 50.74 to − 34.31; p < 0.01) than TG; however, there was no significant difference in the operative time between TG and PG with DTR (WMD − 2.23, CI − 17.67 to 13.21; p = 0.78) or PG with JI (WMD 1.18, CI − 15.42 to 17.77; p = 0.89) (Fig. 2a). Intraoperative blood loss was lower in PG with EG (WMD − 73.65, CI − 109.39 to − 37.90; p < 0.01) or PG with JI (WMD − 38.49, CI − 74.59 to − 2.39; p = 0.04) than in TG; however, there was no significant difference in intraoperative blood loss between PG with DTR and TG (WMD − 2.81, CI − 39.29 to 33.67; p = 0.88) (Fig. 2b). In addition, the duration of postoperative hospital stay was not significantly different between TG and PG with DTR (WMD − 0.67, CI − 2.00 to 0.67; p = 0.33), PG with EG (WMD 1.13, CI − 5.59 to 7.85; p = 0.74), or PG with JI (WMD − 1.59, CI − 4.70 to 1.52; p = 0.32) (Fig. 2c).

In the analyses of postoperative complications, anastomotic leakage and stenosis were included in postoperative complications while gastroesophageal reflux was excluded. No significant difference was found between TG and PG with DTR (OR 0.76, CI 0.45–1.27; p = 0.29), PG with EG (OR 0.96, CI 0.44–2.07; p = 0.91), or PG with JI (OR 0.67, CI 0.42–1.07; p = 0.10) regarding postoperative complications (Fig. 2d). Specifically, there was no significant difference between TG and PG with DTR (OR 0.58, CI 0.32–1.05; p = 0.07), PG with EG (OR 0.60, CI 0.33–1.10; p = 0.10), and PG with JI (OR 0.69, CI 0.19–2.51; p = 0.57) in terms of early complications. Additionally, there was no significant difference between TG and PG with DTR (OR 0.55, CI 0.23–1.27; p = 0.16), PG with EG (OR 1.91, CI 0.49–7.50; p = 0.35), and PG with JI (OR 0.58, CI 0.25–1.37; p = 0.21) regarding late complications (see Supplementary Fig. S2 online). Severe complications (C–D grade ≥ III) occurred less frequently in PG with DTR than in TG (OR 0.42, CI 0.18–0.98; p = 0.05); however, there was no significant difference between TG and PG with DTR regarding less severe complications (C–D grade ≥ I, OR 0.80, CI 0.32–2.05; p = 0.65; and C–D grade ≥ II, OR 0.78, CI 0.46–1.31; p = 0.34) (see Supplementary Fig. S3 online).

In the analyses of operative outcomes, the included studies were subdivided into the laparoscopic and open surgery groups according to the types of PG and TG performed in each study. No studies compared open TG with open PG with DTR. The operative time was shorter in laparoscopic (WMD − 37.92, CI − 48.07 to − 27.76; p < 0.01) and open (WMD − 53.00, CI − 81.31 to − 24.69; p < 0.01) PG with EG compared to laparoscopic and open PG, respectively. Additionally, intraoperative blood loss was lower in laparoscopic PG with EG (WMD − 59.61, CI − 85.83 to − 33.39; p < 0.01) than in laparoscopic TG. However, there was no significant difference between open PG with EG (WMD − 217.62, CI − 525.66 to 90.43; p = 0.17) and open TG. All results of subgroup analyses of operative outcomes subdivided into the laparoscopic and open surgery groups are summarized in Supplementary Table S2 online.

### Postoperative nutritional outcomes

**Figure 3:**
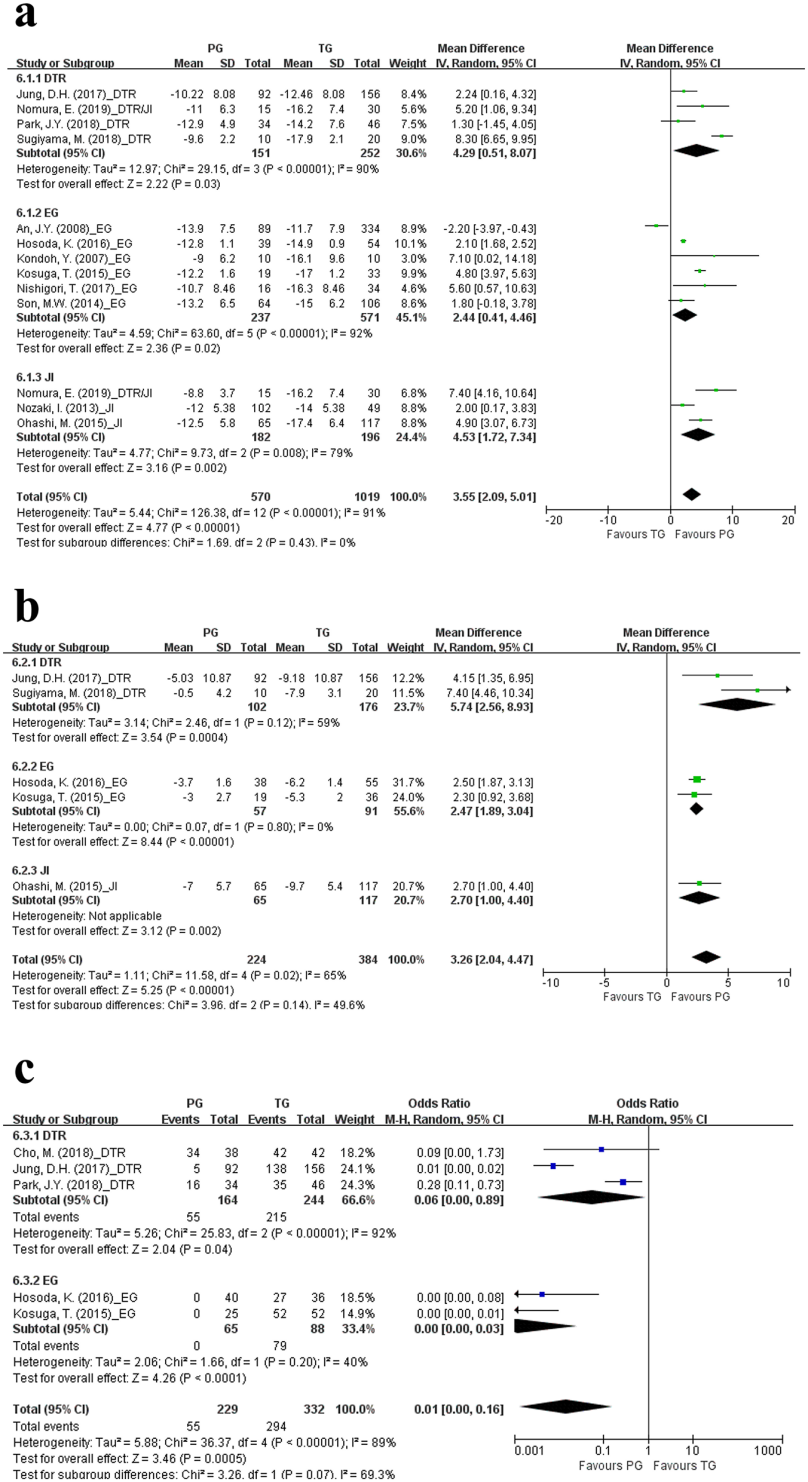
Forest plots for comparing postoperative nutritional outcomes between proximal gastrectomy and total gastrectomy including (**a**) weight change, (**b**) hemoglobin change at postoperative 1 year, and (**c**) postoperative vitamin B_12_ supplementation requirement. The meta-analyses were performed using the Mantel–Haenszel random-effect model. Weighted mean differences or odds ratios are shown with 95% confidence intervals. PG, proximal gastrectomy; TG, total gastrectomy; DTR, double-tract reconstruction; EG, esophagogastrostomy; JI, jejunal interposition.

The analyses of postoperative nutritional outcomes included postoperative weight change, change in hemoglobin level, and postoperative requirement for vitamin B_12_ supplementation. PG was subdivided into the PG with DTR, PG with EG, and PG with JI subgroups in these analyses. Patients who underwent PG with DTR (WMD 4.29, CI 0.51–8.07; p = 0.03), PG with EG (WMD 2.44, CI 0.41–4.46; p = 0.02), and PG with JI (WMD 4.53, CI 1.72–7.34; p < 0.01) had lesser reduction in weight 1 year postoperatively than patients underwent TG (Fig. 3a). In addition, patients who underwent PG with DTR (WMD 5.74, CI 2.56–8.93; p < 0.01), PG with EG (WMD 2.47, CI 1.89–3.04; p < 0.01), and PG with JI (WMD 2.70, CI 1.00–4.40; p < 0.01) had lower rates of hemoglobin reduction 1 year postoperatively than patients underwent TG (Fig. 3b). Moreover, postoperative vitamin B_12_ supplementation was required in fewer patients after PG with DTR (OR 0.06, CI 0.00–0.89; p = 0.04) and PG with EG (OR 0.00, CI 0.00–0.03; p < 0.01) compared to TG (Fig. 3c). Three studies stated that vitamin B_12_ supplementation was provided to patients whose serum vitamin B_12_ levels < 200 pg/mL^33,34,36^. The criterion for vitamin B_12_ supplementation was serum vitamin B_12_ level < 180 pg/mL in the study by Hosoda et al.; however, a definite criterion was not mentioned in the study by Kosuga *et al*^16,42^.

### Complications related to postoperative quality of life

**Figure 4:**
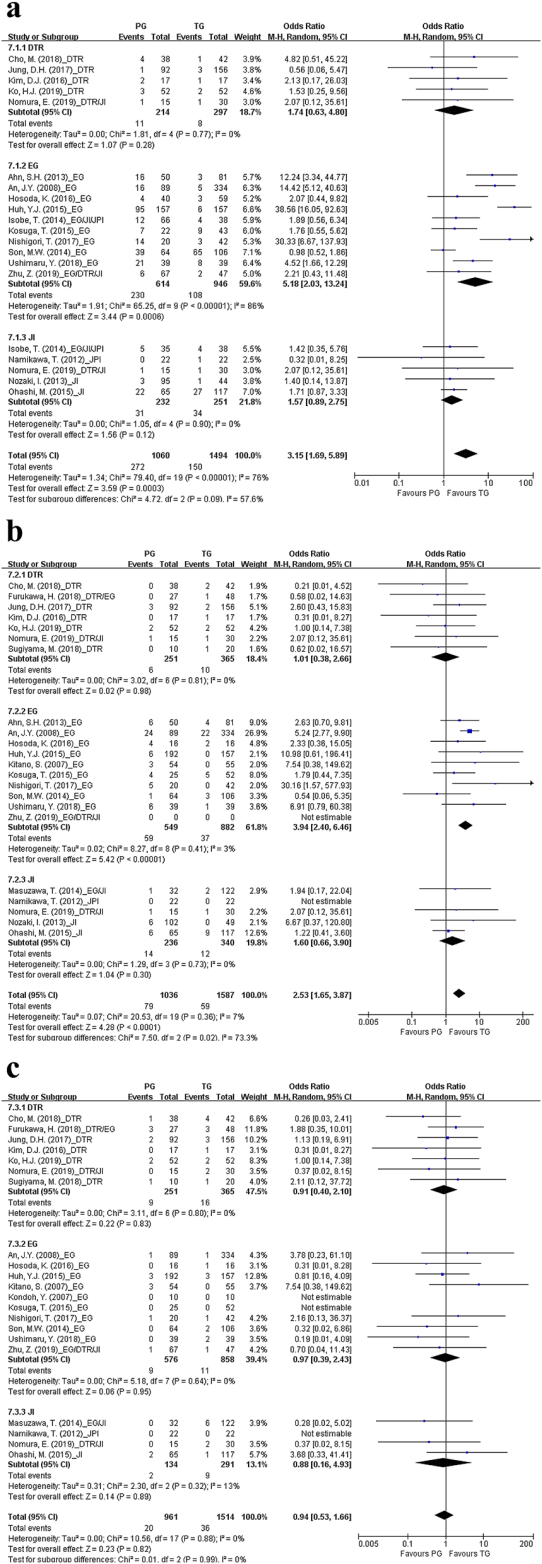
Forest plots for comparing quality of life-related complications between proximal gastrectomy and total gastrectomy including (**a**) gastroesophageal reflux, (**b**) anastomotic stenosis, and (**c**) anastomotic leakage. The meta-analyses were performed using the Mantel–Haenszel random-effect model. Odds ratios are shown with 95% confidence intervals. PG, proximal gastrectomy; TG, total gastrectomy; DTR, double-tract reconstruction; EG, esophagogastrostomy; JI, jejunal interposition; JPI, jejunal pouch interposition.

The analyses of the complications related to postoperative quality of life consisted of three parts: gastroesophageal reflux, anastomotic stenosis, and anastomotic leakage. PG was subdivided into three subgroups: PG with DTR, PG with EG, and PG with JI. In terms of gastroesophageal reflux, TG and PG with DTR (OR 1.74, CI 0.63–4.80; p = 0.28) or PG with JI (OR 1.57, CI 0.89–2.75; p = 0.12) were comparable. However, PG with EG was more likely to cause gastroesophageal reflux than TG (OR 5.18, CI 2.03–13.24; p < 0.01) (Fig. 4a). To summarize, only PG with EG was more likely to cause reflux symptoms than TG (OR 5.91, CI 1.81–19.30; p < 0.01). Similarly, reflux esophagitis, which was confirmed endoscopically, was more likely to occur in PG with EG than in TG (OR 4.22, CI 1.43–12.40; p < 0.01) (see Supplementary Fig. S4 online).

In addition, the occurrence of anastomotic stenosis was similar between PG with DTR (OR 1.01, CI 0.38–2.66; p = 0.98) or PG with JI (OR 1.60, CI 0.66–3.90; p = 0.30) and TG; however, PG with EG was more likely to cause anastomotic stenosis than TG (OR 3.94, CI 2.40–6.46; p < 0.01) (Fig. 4b). Finally, there was no significant difference in the occurrence of anastomotic leakage between TG with PG with DTR (OR 0.91, CI 0.40–2.10; p = 0.83), PG with EG (OR 0.97, CI 0.39–2.43; p = 0.95), and PG with JI (OR 0.88; CI 0.16–4.93; p = 0.89) (Fig. 4c). All results are summarized in Supplementary Table S3. All results of subgroup analyses of postoperative complications and gastroesophageal reflux according to the onset and severity of postoperative complications and the reported type and severity of gastroesophageal reflux, respectively, are summarized in Supplementary Table S4 online.

## Discussion

This study presents an updated summary of evidence regarding the comparison between PG and TG in the treatment of early gastric cancer. Because of the scarcity of prospective or randomized controlled studies on this topic, only retrospective studies were included, limiting the evidence of the study. However, to the best of our knowledge, this study provides clinicians with a detailed comparison between PG and TG regarding operative, nutritional, and postoperative aspects considering various anastomosis types in PG. Patients who underwent PG with EG or PG with DTR had lower weight loss, hemoglobin loss, and postoperative vitamin B_12_ supplementation requirement than those who underwent TG, signifying the nutritional benefit of PG with EG or PG with DTR. Although PG with EG had superior operative outcomes, including reduction in operative time and intraoperative blood loss, more postoperative gastroesophageal reflux and anastomotic leakage were reported after PG with EG than after TG. Therefore, PG with DTR was found to be the most beneficial for the treatment of early gastric cancer.

The surgical safety profiles were similar between PG with DTR and TG, including a shorter operative time, reduced intraoperative blood loss, a shorter postoperative hospital stay, and reduced postoperative complications. DTR is considered a relatively demanding technique because it takes more time to perform additional gastrojejunostomy compared with Roux-en-Y reconstruction. However, both PG with DTR and TG had comparable operation time because suprapyloric and infrapyloric lymph node dissections were usually performed in the latter while mostly reserved in the former. The safety of PG in EGC has been a concern owing to the limited lymph node dissection and the remnant stomach. However, the survival and recurrence rates after PG were reportedly similar to that after TG in recent meta-analyses^53,54^. In subgroup analyses according to open and laparoscopic approaches, PG with EG had a shorter operative time than TG, which could be attributable to its relatively less complicated surgical procedure in both laparoscopic and open approaches. Patients who underwent laparoscopic PG with EG experienced lesser intraoperative blood loss than those who underwent laparoscopic TG, which could be presumed based on the smallest operation range and the simplest anastomotic procedure among laparoscopic PG with the three anastomosis types and TG.

Furthermore, PG with DTR had less severe postoperative complications (C–D score ≥ III) than TG; this result is inconsistent with that reported in a previous study^53^, in which there was no significant difference between PG with DTR and TG with regard to postoperative complications. This inconsistency seems to have been caused by the fact that our meta-analysis included more studies. Ko et al. reported that 3 patients with TG experienced pancreatic fistula, while no patients who underwent PG with DTR experienced pancreatic fistula^32^. The occurrence of pancreatic fistula after TG can be attributed to infrapyloric lymph node dissection, which results in pancreatic injury^55^. Although infrapyloric lymph node dissection is usually performed in TG to reduce the likelihood of recurrence and metastasis, radical dissection of peripancreatic lymph nodes can lead to severe complications related to pancreatic injury.

Regarding postoperative nutritional outcomes, the present meta-analysis revealed that patients who underwent PG with DTR had lower weight loss, hemoglobin loss, and postoperative vitamin B_12_ supplementation requirement than those who underwent TG, which is consistent with the findings of previous meta-analyses^53,54^. The difference in the severity of weight loss is attributable to food passage through the duodenum in DTR. The preservation of duodenal food passage has physiological benefits in nutrient absorption with adequate chyme with bile and pancreatic juice and gastrointestinal hormonal regulation^56^. Hence, patients without duodenal food passage after undergoing PG with DTR experienced more weight loss in a previous study^57^. These nutritional characteristics could be beneficial for patients with cancer because weight loss is associated with shorter failure-free and overall survival and decreased response to chemotherapy, quality of life, and performance status. Given that patients with EGC do not receive chemotherapy, weight loss is one of the important factors determining the quality of life in patients with cancer^58^. In particular, more than 10% of weight loss after gastrectomy was related to disabling symptoms and had a negative impact on the quality of life^59^.

Additionally, patients who underwent PG were less likely to have vitamin B_12_ deficiency, which can be attributed to the remnant stomach where the intrinsic factor from parietal cells absorb vitamin B_12_^60^. Given that vitamin B_12_ deficiency is known to cause megaloblastic anemia and neuropsychiatric symptoms, the advantage of PG in ensuring vitamin B_12_ absorption has a clinically beneficial impact on the quality of life of patients^8^. Similarly, lower hemoglobin loss in PG than in TG 1 year after gastrectomy is likely to be related to the lower incidence of vitamin B_12_ deficiency in patients who underwent PG in the present study. However, previous studies reported that anemia, which occurs during the first few years after TG is mainly caused by iron deficiency, rather than vitamin B_12_ deficiency, because it takes a few years to deplete the human body’s stores of vitamin B_12_^61,62^. Future studies with long-term follow-up are needed to elucidate the different underlying mechanisms of anemia in patients who underwent PG or TG.

PG with DTR was also similar to TG in terms of postoperative complications related to the quality of life. Specifically, the incidence of gastroesophageal reflux or anastomotic stenosis was similar between PG with DTR or PG with JI and TG; however, the incidence was higher in PG with EG than in TG. The long Roux-en-Y limb functions as a barrier to the reflux in TG with Roux-en-Y reconstruction, while the mechanisms to prevent reflux are less effective in PG with EG^10,63^. The jejunal limb of DTR is also thought to have a similar function to the Roux-en-Y limb in TG, contributing to the lesser occurrence of reflux. Moreover, there was no difference in anastomotic leakage between TG and PG with DTR. Considering that DTR has 2 more anastomotic sites than EG, this result implicates the feasibility of DTR despite it being a more complicated surgical procedure. Hence, PG with DTR can be considered relatively safe to maintain an acceptable quality of life after surgery.

Among the three anastomosis types of PG, EG and JI have proved to be superior to TG in a few operative outcomes, such as operation time (EG) and intraoperative blood loss (EG and JI). However, the results of postoperative complications related to anastomosis and its symptoms differed depending on the specific type of anastomosis. When PG was performed with EG, anastomotic stenosis and gastroesophageal reflux occurred more frequently than in TG, leading to the conclusion that it is not an optimal treatment method for EGC. In contrast, there was no significant difference between PG with JI and TG in terms of anastomotic stenosis and reflux due to the function of JI as an alternative sphincter. However, PG with JI is not generally performed due to its technical difficulty during laparoscopic procedures^64^.

To overcome the limitations of EG like symptomatic reflux, several additional anti-reflux techniques have been designed, such as the double flap technique (DFT) during EG with valvuloplasty, in which a seromuscular flap is created to avoid reflux^18,19^. In a previous retrospective study performed by Hayami et al*.*, anastomotic complications (4.7% vs. 17.2%) and severe reflux esophagitis with LA grade ≥ B (2.3% vs. 14.9%) occurred less frequently after EG with DFT than after TG, but the difference was not significant (p = 0.093 and p = 0.06, respectively)^19^. Although EG with DFT was excluded from the present meta-analysis because of insufficient data and the heterogeneity of EG combined with DFT due to EG in operative and reflux-related outcomes, the efficacy and safety of this technique should also be evaluated by comparing EG with DFT and other methods in future studies^18,19,65^.

In the bias assessment using the ROBINS-I tool, two studies were identified to have serious biases. In one study, the bias was due to the selection of the reported results regarding postoperative complications, while in the other study, the bias was due to the inconsistent frequency of postoperative upper endoscopic examination between PG and TG^46,48^. In the present meta-analysis, the studies by Furukawa et al. and Zhou et al., including multiple types of anastomosis in PG without classification, were classified into subgroups, with the most common type accounting for more than 75% of the overall cohort, which might cause bias. In this reason, additional analyses excluding the studies by Furukawa et al*.* and Zhou et al*.* were conducted to minimize heterogeneity and bias. In the analysis, there was no difference in the tendency of preference between PG and TG in all aspects. In addition, the studies by Nomura et al. and Isobe et al., comparing TG with PG with two different types of anastomosis, were classified into both subgroups, which might also increase bias because the events in the TG group would be repeatedly calculated in the process of synthesizing all subgroup analyses. Therefore, we performed additional analyses in which the events in the TG and PG groups were counted only once regardless of the number of anastomosis type in the study by combining the data from each anastomosis type. In the analysis, there was also no difference in the tendency of preference between PG and TG in all aspects. The results of additional analyses are provided in Supplementary Table S5 and S6 online.

Our meta-analysis has several limitations. First, there is a lack of prospective cohort studies and randomized controlled trials. Although four case-control studies were included, there is a possibility of selection and confounding bias. Although no publication bias was identified in the symmetry of funnel plots, it was difficult to exclude publication bias because other biases accompanying publication bias could not be excluded in the analyses of retrospective studies^66^. Second, both JI and JPI were included in the JI subgroup because of their similarity and the smaller number of studies evaluating them. Finally, although the present meta-analysis revealed a tendency of preference for PG in terms of hemoglobin change and vitamin B_12_ deficiency, the number of studies included in the analyses of hemoglobin change 1 year postoperatively and postoperative vitamin B_12_ supplementation requirement was insufficient to provide reliability. Considering that only a limited number of retrospective studies have been performed thus far, further studies are warranted to elucidate the metabolic impact of PG and TG. In addition, randomized controlled trials including comparison of nutritional outcomes between PG and TG and analyses comparing PG with JI and PG with JPI separately should be conducted to accumulate evidence and minimize bias.

In summary, PG with DTR is superior to TG in terms of nutritional outcomes, causing lesser weight reduction, and reduced hemoglobin loss, with reduced postoperative vitamin B_12_ supplementation requirement. Furthermore, PG with DTR has no disadvantage regarding operative outcomes and the postoperative complications related to quality of life such as gastroesophageal reflux and anastomotic leakage. In conclusion, PG with DTR can be a promising treatment option for patients with EGC without compromising nutritional requirements and postoperative quality of life. Considering the superiority of PG with EG regarding operative outcomes, the safety and efficacy of EG with anti-reflux procedures such as DFT should be evaluated in future studies.

## Supplementary information

Supplementary information.

## Data Availability

All data relevant to the present study are provided in the manuscript and supplementary materials.
