# Supplementary material for: Postoperative nutritional outcomes and quality of life-related complications of proximal versus total gastrectomy for upper-third early gastric cancer: a meta-analysis
Source: Sci Rep. 2020 Dec 8;10:21460. doi: 10.1038/s41598-020-78458-0 (PMC7722732; doi:10.1038/s41598-020-78458-0)
Supplement: Supplementary file 1 — Supplementary information. [file 41598_2020_78458_MOESM1_ESM.pdf]

Postoperative nutritional outcomes and quality of life-related complications of proximal *versus* total gastrectomy for upper-third early gastric cancer: a meta-analysis

Inhyeok Lee[1†]; Youjin Oh[1†]; Shin-Hoo Park[2]; Yeongkeun Kwon[2]; Sungsoo Park[2*]

[1] Department of Medicine, Korea University College of Medicine, Seoul, Republic of Korea.

[2] Division of Foregut Surgery, Korea University College of Medicine, Seoul, Republic of Korea.

† These authors contributed equally to this work.

Study (publish year)	Surgery type	N	BMI		
			<25	25-30	>30
Kitano, S., et al. (2007)	PG	54	40	13	1
	TG	55	52	2	1

Supplementary Table S1. Summary of the body mass index group in a study included in this meta-analysis. PG, proximal gastrectomy; TG, total gastrectomy; BMI, body mass index.

Outcome		Comparison				Heterogeneity		Favour
		n	WMD (95% CI)	OR (95% CI)	*p*	Tau^2	I^2	
**1. Operation time (min)**								
Laparoscopic surgery		11	-18.73 (-33.75 to -3.70)		<0.01	511.83	85%	PG
	PG with DTR	6	-1.55 (-22.58 to 19.49)		0.89	550.73	83%	NSD
	PG with EG	5	-37.92 (-48.07 to -27.76)		<0.01	42.56	34%	EG
	PG with JI	1	-19.40 (-41.47 to 2.67)		0.08	-	-	NSD
Open surgery		3	-25.63 (-86.31 to 35.05)		0.41	2555.93	91%	NSD
	PG with EG	2	-53.00 (-81.31 to -24.69)		<0.01	0.00	0%	EG
	PG with JI	1	25.00 (7.52 to 42.48)		<0.01	-	-	TG
**2. Intraoperative blood loss (mL)**								
Laparoscopic surgery		9	-25.25 (-59.72 to 9.22)		0.15	2215.1	85%	NSD
	PG with DTR	5	6.73 (-42.79 to 56.25)		0.79	2543.65	82%	NSD
	PG with EG	4	-59.61 (-85.83 to -33.39)		<0.01	316.91	46%	EG
	PG with JI	1	-39.30 (-87.29 to 8.69)		0.11	-	-	NSD
Open surgery		3	-118.32 (-332.33 to 95.69)		0.28	22414.94	63%	NSD
	PG with EG	2	-217.62 (-525.66 to 90.43)		0.17	24885.12	50%	NSD
	PG with JI	1	-15.00 (-98.81 to 68.81)		0.73	-	-	NSD
**3. Postoperative hospital stay (days)**								
Laparoscopic surgery		8	-0.44 (-3.03 to 2.16)		0.74	9.78	82%	NSD
	PG with DTR	5	-0.36 (-1.74 to 1.03)		0.61	0.69	28%	NSD
	PG with EG	3	5.82 (-8.49 to 20.13)		0.43	140.39	94%	NSD
	PG with JI	1	-4.60 (-9.31 to 0.11)		0.06	-	-	NSD
Open surgery		1	0.00 (-5.51 to 5.51)		1.00	-	-	NSD
	PG with JI	1	0.00 (-5.51 to 5.51)		1.00	-	-	NSD
**4. Postoperative complications**								
Laparoscopic surgery		11		1.09 (0.67 to 1.77)	0.73	0.31	51%	NSD
	PG with DTR	6		0.97 (0.48 to 1.97)	0.94	0.35	47%	NSD
	PG with EG	5		1.29 (0.64 to 2.61)	0.48	0.34	53%	NSD
	PG with JI	1		0.42 (0.08 to 2.30)	0.32	-	-	NSD
Open surgery		4		0.77 (0.45 to 1.30)	0.33	0.00	0%	NSD
	PG with EG	2		0.59 (0.22 to 1.57)	0.29	0.18	36%	NSD
	PG with JI	1		0.93 (0.39 to 1.66)	0.86	-	-	NSD

Supplementary Table S2. Results of the subgroup analyses of operative outcomes subdivided according to the type of surgery. All analyses were performed using a random-effects model. WMD, weighted mean difference; OR, odds ratio; PG, proximal gastrectomy; TG, total gastrectomy; DTR, double-tract reconstruction; EG, esophagogastrostomy; JI, jejunal interposition; NSD, no significant difference.

Outcome		Comparison				Heterogeneity		Favour
		n	WMD (95% CI)	OR (95% CI)	*p*	Tau^2	I^2	
**1. Operative findings**								
Operation time (min)†‡		21	-17.89 (-29.64 to -6.13)		<0.01	599.27	85%	PG
	PG with DTR	8	-2.23 (-17.67 to 13.21)		0.78	365.10	77%	NSD
	PG with EG	8	-42.53 (-50.74 to -34.31)		<0.01	34.31	26%	EG
	PG with JI	5	1.18 (-15.42 to 17.77)		0.89	216.28	63%	NSD
Intraoperative blood loss (mL)†‡		18	-35.38 (--61.27 to -9.48)		<0.01	1891.22	75%	PG
	PG with DTR	7	-2.81 (-39.29 to 33.67)		0.88	1776.51	75%	NSD
	PG with EG	7	-73.65 (-109.39 to -37.90)		<0.01	981.64	59%	EG
	PG with JI	4	-38.49 (-74.59 to -2.39)		0.04	0.00	0%	JI
Postoperative hospital stay (days)†‡		16	-0.94 (-2.73 to 0.85)		0.30	7.72	70%	NSD
	PG with DTR	7	-0.67 (-2.00 to 0.67)		0.33	0.97	31%	NSD
	PG with EG	5	1.13 (-5.59 to 7.85)		0.74	47.38	89%	NSD
	PG with JI	4	-1.59 (-4.70 to 1.52)		0.32	0.00	0%	NSD
Postoperative complications†‡		23		0.84 (0.55 to 1.30)	0.44	0.78	75%	NSD
	PG with DTR	8		0.76 (0.45 to 1.27)	0.29	0.21	40%	NSD
	PG with EG	11		0.96 (0.44 to 2.07)	0.91	1.37	85%	NSD
	PG with JI	4		0.67 (0.42 to 1.07)	0.10	0.00	0%	NSD
**2. Nutritional parameters**								
Weight change (%) - PO 1 year†		13	3.55 (2.09 to 5.01)		<0.01	5.44	91%	PG
	PG with DTR	4	4.29 (0.51 to 8.07)		0.03	12.97	90%	DTR
	PG with EG	6	2.44 (0.41 to 4.46)		0.02	4.59	92%	EG
	PG with JI	3	4.53 (1.72 to 7.34)		<0.01	4.77	79%	JI
Hemoglobin change (%) - PO 1 year		5	3.26 (2.04 to 4.47)		<0.01	1.11	65%	PG
	PG with DTR	2	5.74 (2.56 to 8.93)		<0.01	3.14	59%	DTR
	PG with EG	2	2.47 (1.89 to 3.04)		<0.01	0.00	0%	EG
	PG with JI	1	2.70 (1.00 to 4.40)		<0.01	-	-	JI
Postoperative vitamin B_{12} supplementation		5		0.01 (0.00 to 0.16)	<0.01	5.88	89%	PG
	PG with DTR	3		0.06 (0.00 to 0.89)	0.04	5.26	92%	DTR
	PG with EG	2		0.00 (0.00 to 0.03)	<0.01	2.06	40%	EG
Postoperative iron supplementation		3		0.47 (0.20 to 1.14)	0.09	0.00	0%	NSD
Serum albumin change (%) - PO 1 year		3	3.69 (0.28 to 7.11)		0.03	7.31	96%	PG
Lymphocyte count change (%) - PO 1 year		3	9.36 (-0.04 to 18.76)		0.05	63.74	95%	NSD
**3, Quality of life related outcomes**								
Gastroesophageal reflux†‡		20		3.15 (1.69 to 5.89)	<0.01	1.34	76%	TG
	PG with DTR	5		1.74 (0.63 to 4.80)	0.28	0.00	0%	NSD
	PG with EG	10		5.18 (2.03 to 13.24)	<0.01	1.91	86%	TG
	PG with JI	5		1.57 (0.89 to 2.75)	0.12	0.00	0%	NSD
Anastomotic stenosis†		22		2.53 (1.65 to 3.87)	<0.01	0.07	7%	TG
	PG with DTR	7		1.01 (0.38 to 2.66)	0.98	0.00	0%	NSD
	PG with EG	10		3.94 (2.40 to 6.46)	<0.01	0.02	3%	TG
	PG with JI	5		1.60 (0.66 to 3.90)	0.30	0.00	0%	NSD
Anastomotic leakage†		21		0.94 (0.53 to 1.66)	0.82	0.00	0%	NSD

PG with DTR	7		0.91 (0.40 to 2.10)	0.83	0.00	0%	NSD
PG with EG	10		0.97 (0.39 to 2.43)	0.95	0.00	0%	NSD
PG with JI	4		0.88 (0.16 to 4.93)	0.89	0.31	13%	NSD

Supplementary Table S3. Results of the meta-analyses. † Analyses with parenthesis including duplicated cohort with total gastrectomy due to their subgroup analyses between PG with DTR or JI and TG in the study by Nomura *et al.* ‡ Analyses with parenthesis including duplicated cohort with total gastrectomy due to their subgroup analyses between PG with EG or JI and TG in the study by Isobe *et al.* All analyses were performed using a random-effects model. WMD, weighted mean difference; OR, odds ratio; PG, proximal gastrectomy; TG, total gastrectomy; DTR, double-tract reconstruction; EG, esophagogastrostomy; JI, jejunal interposition; NSD, no significant difference.

			Comparison			Heterogeneity		
Outcome			*n*	OR (95% CI)	*p*	Tau²	*I*²	Favour
**1. Postoperative complications**								
**Onset of postoperative complications**								
	Early complications		9	0.58 (0.42 to 0.79)	0.97	0.00	0%	PG
		PG with DTR	3	0.58 (0.32 to 1.05)	0.81	0.00	0%	NSD
		PG with EG	5	0.60 (0.33 to 1.10)	0.14	0.19	43%	NSD
		PG with JI	1	0.69 (0.19 to 2.51)	0.49	-	-	NSD
	Late complications		7	1.07 (0.48 to 2.42)	0.87	0.78	69%	NSD
		PG with DTR	2	0.55 (0.23 to 1.27)	0.16	0.00	0%	NSD
		PG with EG	4	1.91 (0.49 to 7.50)	0.35	1.51	78%	NSD
		PG with JI	1	0.58 (0.25 to 1.37)	0.21	-	-	NSD
**Severity of postoperative complications**								
	C-D grade ≥ I		13	0.84 (0.44 to 1.57)	0.58	1.07	83%	NSD
		PG with DTR	4	0.80 (0.32 to 2.05)	0.65	0.58	65%	NSD
		PG with EG	5	1.02 (0.28 to 3.80)	0.97	2.00	91%	NSD
		PG with JI	4	0.67 (0.42 to 1.07)	0.10	0.00	0%	NSD
	C-D grade ≥II		9	0.99 (0.60 to 1.64)	0.97	0.26	46%	NSD
		PG with DTR	5	0.78 (0.46 to 1.31)	0.34	0.07	19%	NSD
		PG with EG	4	1.22 (0.48 to 3.09)	0.67	0.54	61%	NSD
	C-D grade ≥III		5	0.53 (0.26 to 1.08)	0.08	0.00	0%	NSD
		PG with DTR	4	0.42 (0.18 to 0.98)	0.05	0.00	0%	DTR
		PG with JI	1	0.89 (0.26 to 3.09)	0.86	-	-	NSD
**2. Gastroesophageal reflux**								
**Reflux symptoms**			12	2.80 (1.37 to 5.70)	<0.01	0.73	55%	TG
		PG with DTR	3	1.30 (0.29 to 5.78)	0.73	0.00	0%	NSD
		PG with EG	6	5.91 (1.81 to 19.30)	<0.01	1.40	70%	TG
		PG with JI	3	1.56 (0.86 to 2.83)	0.14	0.00	0%	NSD
	Visick score ≥II		11	2.36 (1.29 to 4.33)	<0.01	0.36	39%	TG
		PG with DTR	3	1.30 (0.29 to 5.78)	0.73	0.00	0%	NSD
		PG with EG	5	4.16 (1.45 to 11.96)	<0.01	0.84	62%	TG
		PG with JI	3	1.56 (0.86 to 2.83)	0.14	0.00	0%	NSD
	Visick score ≥III		2	26.77 (4.95 to 144.68)	<0.01	0.00	0%	TG
		PG with EG	2	26.77 (4.95 to 144.68)	<0.01	0.00	0%	TG
**Reflux esophagitis**			11	3.23 (1.37 to 7.61)	<0.01	1.39	75%	TG
		PG with DTR	2	1.27 (0.25 to 6.53)	0.77	0.00	0%	NSD
		PG with EG	7	4.22 (1.43 to 12.40)	<0.01	1.71	84%	TG
		PG with JI	2	1.64 (0.27 to 9.74)	0.59	0.00	0%	NSD
	LA grade ≥C		6	1.16 (0.35 to 3.78)	0.81	0.97	49%	NSD
		PG with DTR	1	0.33 (0.01 to 8.21)	0.5	-	-	NSD
		PG with EG	4	1.39 (0.29 to 6.61)	0.68	1.61	68%	NSD
		PG with JI	1	1.41 (0.06 to 35.37)	0.83	-	-	NSD

Supplementary Table S4. Results of the subgroup analyses of postoperative complications and gastroesophageal reflux according to the onset and severity of postoperative complications and the reported

type and severity of gastroesophageal reflux. All analyses were performed using a random-effects model. OR, odds ratio; PG, proximal gastrectomy; TG, total gastrectomy; DTR, double-tract reconstruction; EG, esophagogastrostomy; JI, jejunal interposition; NSD, no significant difference.

		Comparison				Heterogeneity		
Outcome		*n*	WMD (95% CI)	OR (95% CI)	*p*	Tau^2	I^2	Favour
**1. Operative findings**								
Operation time (min)†		20	-20.35 (-31.61 to -9.09)		<0.01	503.62	83%	PG
	PG with DTR	7	-7.70 (-21.20 to 5.80)		0.26	202.34	65%	NSD
	PG with EG	8	-42.53 (-50.74 to -34.31)		<0.01	34.31	26%	EG
	PG with JI	5	1.18 (-15.42 to 17.77)		0.89	216.28	63%	NSD
Intraoperative blood loss (mL)†		17	-39.42 (-65.74 to -13.10)		<0.01	1812.96	74%	PG
	PG with DTR	6	-7.55 (--48.07 to 32.97)		0.72	1962.08	78%	NSD
	PG with EG	7	-73.65 (-109.39 to -37.90)		<0.01	981.64	59%	EG
	PG with JI	4	-38.49 (-74.59 to -2.39)		0.04	0.00	0%	JI
Postoperative hospital stay (days)		16	-0.94 (-2.73 to 0.85)		0.30	7.72	70%	NSD
	PG with DTR	7	-0.67 (-2.00 to 0.67)		0.33	0.97	31%	NSD
	PG with EG	5	1.13 (-5.59 to 7.85)		0.74	47.38	89%	NSD
	PG with JI	4	-1.59 (-4.70 to 1.52)		0.32	0.00	0%	NSD
Postoperative complications†‡		21		0.85 (0.54 to 1.35)	0.50	0.80	76%	NSD
	PG with DTR	8		0.68 (0.40 to 1.15)	0.15	0.17	36%	NSD
	PG with EG	11		0.96 (0.44 to 2.07)	0.91	1.37	85%	NSD
	PG with JI	4		0.67 (0.42 to 1.07)	0.10	0.00	0%	NSD
**2. Nutritional parameters**								
Weight change (%) - PO 1 year		13	3.55 (2.09 to 5.01)		<0.01	5.44	91%	PG
	PG with DTR	4	4.29 (0.51 to 8.07)		0.03	12.97	90%	DTR
	PG with EG	6	2.44 (0.41 to 4.46)		0.02	4.59	92%	EG
	PG with JI	3	4.53 (1.72 to 7.34)		<0.01	4.77	79%	JI
Hemoglobin change (%) - PO 1 year		5	3.26 (2.04 to 4.47)		<0.01	1.11	65%	PG
	PG with DTR	2	5.74 (2.56 to 8.93)		<0.01	3.14	59%	DTR
	PG with EG	2	2.47 (1.89 to 3.04)		<0.01	0.00	0%	EG
	PG with JI	1	2.70 (1.00 to 4.40)		<0.01	-	-	JI
Postoperative vitamin B12 supplementation		5		0.01 (0.00 to 0.16)	<0.01	5.88	89%	PG
	PG with DTR	3		0.06 (0.00 to 0.89)	0.04	5.26	92%	DTR
	PG with EG	2		0.00 (0.00 to 0.03)	<0.01	2.06	40%	EG
Postoperative iron supplementation		3		0.47 (0.20 to 1.14)	0.09	0.00	0%	NSD
Serum albumin change (%) - PO 1 year		3	3.69 (0.28 to 7.11)		0.03	7.31	96%	PG
Lymphocyte count change (%) - PO 1 year		3	9.36 (-0.04 to 18.76)		0.05	63.74	95%	NSD
**3, Quality of life related outcomes**								
Gastroesophageal reflux‡		19		3.20 (1.67 to 6.15)	<0.01	1.40	77%	TG
	PG with DTR	5		1.74 (0.63 to 4.80)	0.28	0.00	0%	NSD
	PG with EG	9		5.63 (2.05 to 15.42)	<0.01	2.03	88%	TG
	PG with JI	5		1.57 (0.89 to 2.75)	0.12	0.00	0%	NSD
Anastomotic stenosis†‡		20		2.59 (1.68 to 3.98)	<0.01	0.07	8%	TG
	PG with DTR	6		1.07 (0.39 to 2.94)	0.98	0.00	0%	NSD
	PG with EG	9		3.94 (2.40 to 6.46)	<0.01	0.02	3%	TG
	PG with JI	5		1.60 (0.66 to 3.90)	0.30	0.00	0%	NSD
Anastomotic leakage†‡		21		0.94 (0.53 to 1.66)	0.82	0.00	0%	NSD

PG with DTR	6		0.72 (0.27 to 1.88)	0.50	0.00	0%	NSD
PG with EG	9		1.01 (0.38 to 2.67)	0.98	0.00	0%	NSD
PG with JI	4		0.88 (0.16 to 4.93)	0.89	0.31	13%	NSD

Supplementary Table S5. Results of the meta-analyses excluding the studies by Furukawa *et al.* and Zhou *et al.*. † Analyses with parenthesis including duplicated cohort with total gastrectomy due to their subgroup analyses between PG with DTR or JI and TG in the study by Nomura *et al.*; ‡ Analyses with parenthesis including duplicated cohort with total gastrectomy due to their subgroup analyses between PG with EG or JI and TG in the study by Isobe *et al.*. All analyses were performed using a random-effects model. WMD, weighted mean difference; OR, odds ratio; PG, proximal gastrectomy; TG, total gastrectomy; DTR, double-tract reconstruction; EG, esophagogastrostomy; JI, jejunal interposition; NSD, no significant difference.

Outcome	Comparison *n*	WMD (95% CI)	OR (95% CI)	*p*	Heterogeneity Tau^2	I^2	Favour
**1. Operative findings**							
Operation time (min)†‡	19	-17.89 (-30.02 to -5.77)		<0.01	578.90	86%	PG
Intraoperative blood loss (mL)†‡	16	-30.83 (-57.21 to -4.45)		0.02	1760.97	75%	PG
Postoperative hospital stay (days)†‡	14	-0.58 (-2.40 to 1.23)		0.53	6.96	70%	NSD
Postoperative complications†‡	21		0.89 (0.57 to 1.39)	0.60	0.77	76%	NSD
**2. Nutritional parameters**							
Weight change (%) - PO 1 year†	12	3.37 (1.88 to 4.87)		<0.01	5.35	91%	PG
Hemoglobin change (%) - PO 1 year	5	3.26 (2.04 to 4.47)		<0.01	1.11	65%	PG
Postoperative vitamin B_{12} supplementation	5		0.01 (0.00 to 0.16)	<0.01	5.88	89%	PG
Postoperative iron supplementation	3		0.47 (0.20 to 1.14)	0.09	0.00	0%	NSD
Serum albumin change (%) - PO 1 year	3	3.69 (0.28 to 7.11)		0.03	7.31	96%	PG
Lymphocyte count change (%) - PO 1 year	3	9.36 (-0.04 to 18.76)		0.05	63.74	95%	NSD
**3, Quality of life related outcomes**							
Gastroesophageal reflux†‡	18		3.28 (1.68 to 6.40)	<0.01	1.44	78%	TG
Anastomotic stenosis†	21		2.47 (1.57 to 3.87)	<0.01	0.11	12%	TG
Anastomotic leakage†	20		0.94 (0.52 to 1.70)	0.84	0.00	0%	NSD

Supplementary Table S6. Results of the meta-analyses without repeatedly calculated events in the TG group of the studies by Nomura *et al.* and Isobe *et al.* † Analyses including the study by Nomura *et al.*; ‡ Analyses including the study by Isobe *et al.*; All analyses were performed using a random-effects model; WMD, weighted mean difference; OR, odds ratio; PG, proximal gastrectomy; TG, total gastrectomy; NSD, no significant difference.

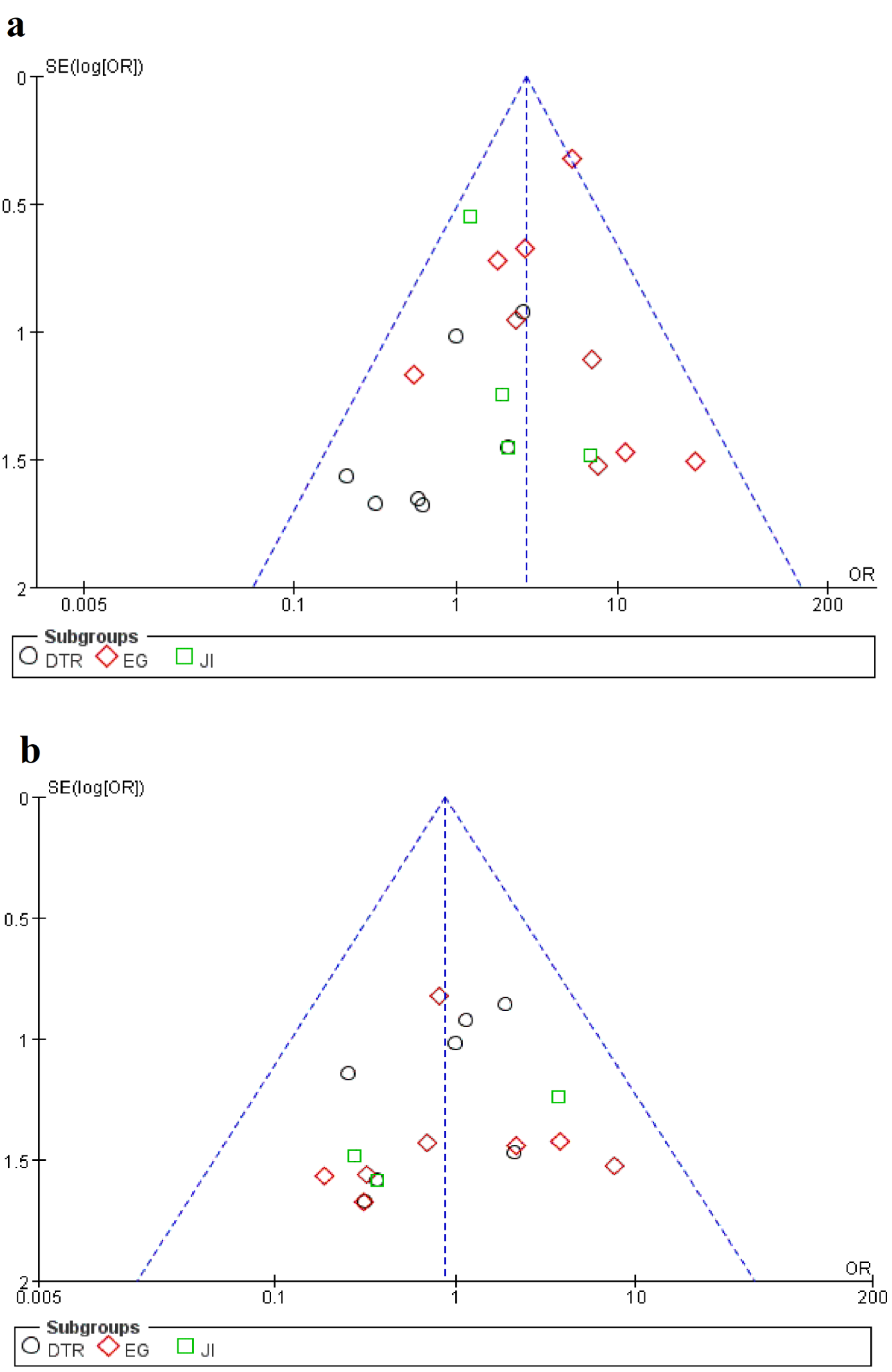

Supplementary Figure S1. Funnel plots for meta-analyses of **a)** anastomotic stenosis and **b)** anastomotic leakage. DTR, double-tract reconstruction; EG, esophagogastrostomy; JI, jejunal interposition.

a

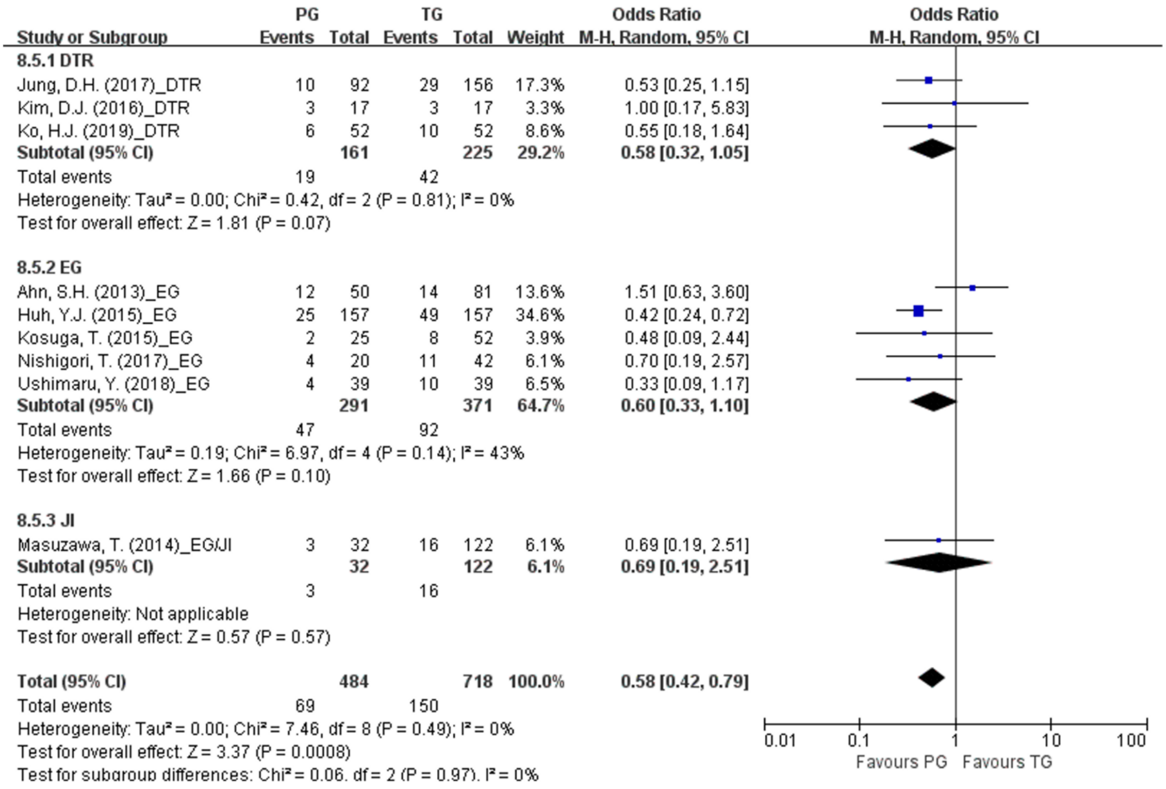

b

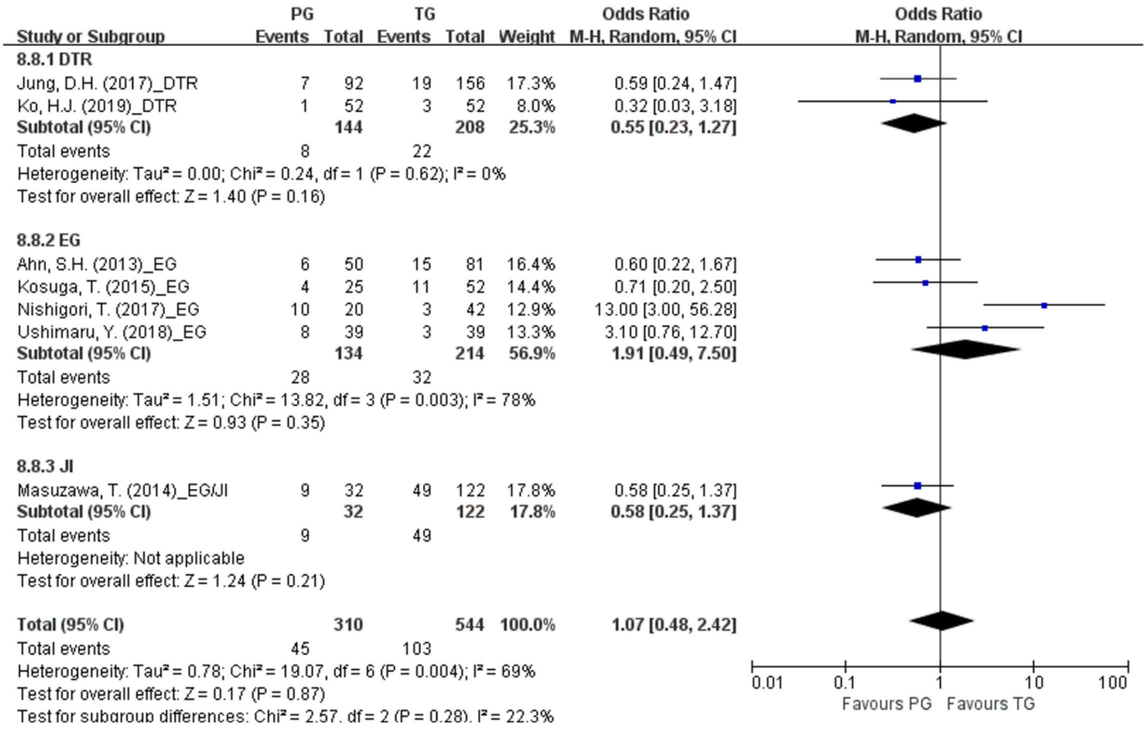

Supplementary Figure S2. Forest plots for comparing **a)** early complications and **b)** late complications between proximal gastrectomy and total gastrectomy. The meta-analysis was performed with the Mantel–Haenszel random-effects model. Odds ratios are shown with 95% confidence intervals. PG, proximal gastrectomy; TG, total gastrectomy; DTR, double-tract reconstruction; EG, esophagogastrostomy; JI, jejunal interposition.

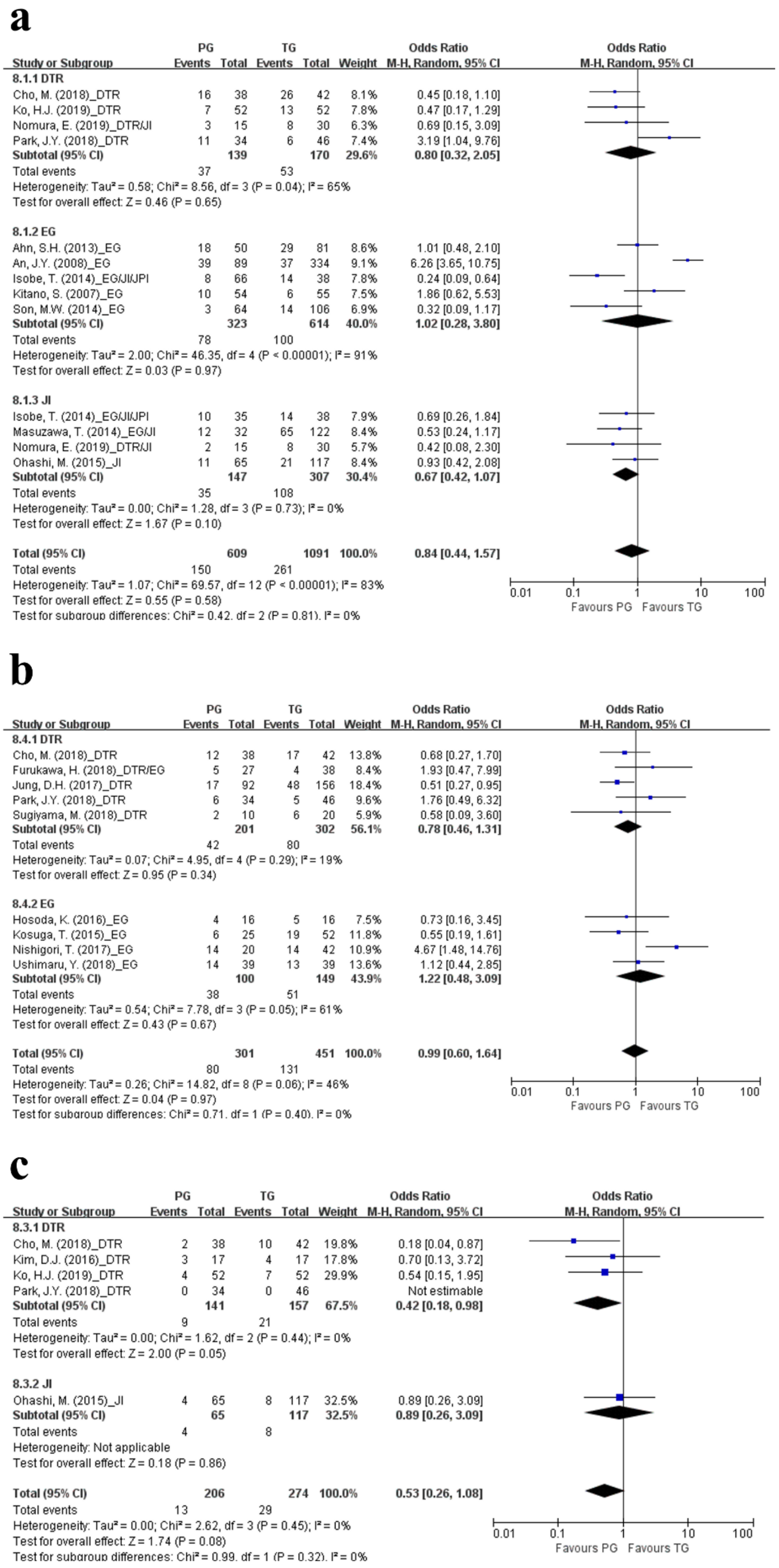

Supplementary Figure S3. Forest plots for comparing postoperative complications classified by the severity of the complications between proximal gastrectomy and total gastrectomy including **a)** C-D grade ≥ I, **b)** C-D grade ≥ II, and **c)** C-D grade ≥ III. The meta-analysis was performed with the Mantel–Haenszel random-effects model. Odds ratios are shown with 95% confidence intervals. PG, proximal gastrectomy; TG, total gastrectomy; DTR, double-tract reconstruction; EG, esophagogastrostomy; JI, jejunal interposition; C-D, Clavien–Dindo classification.

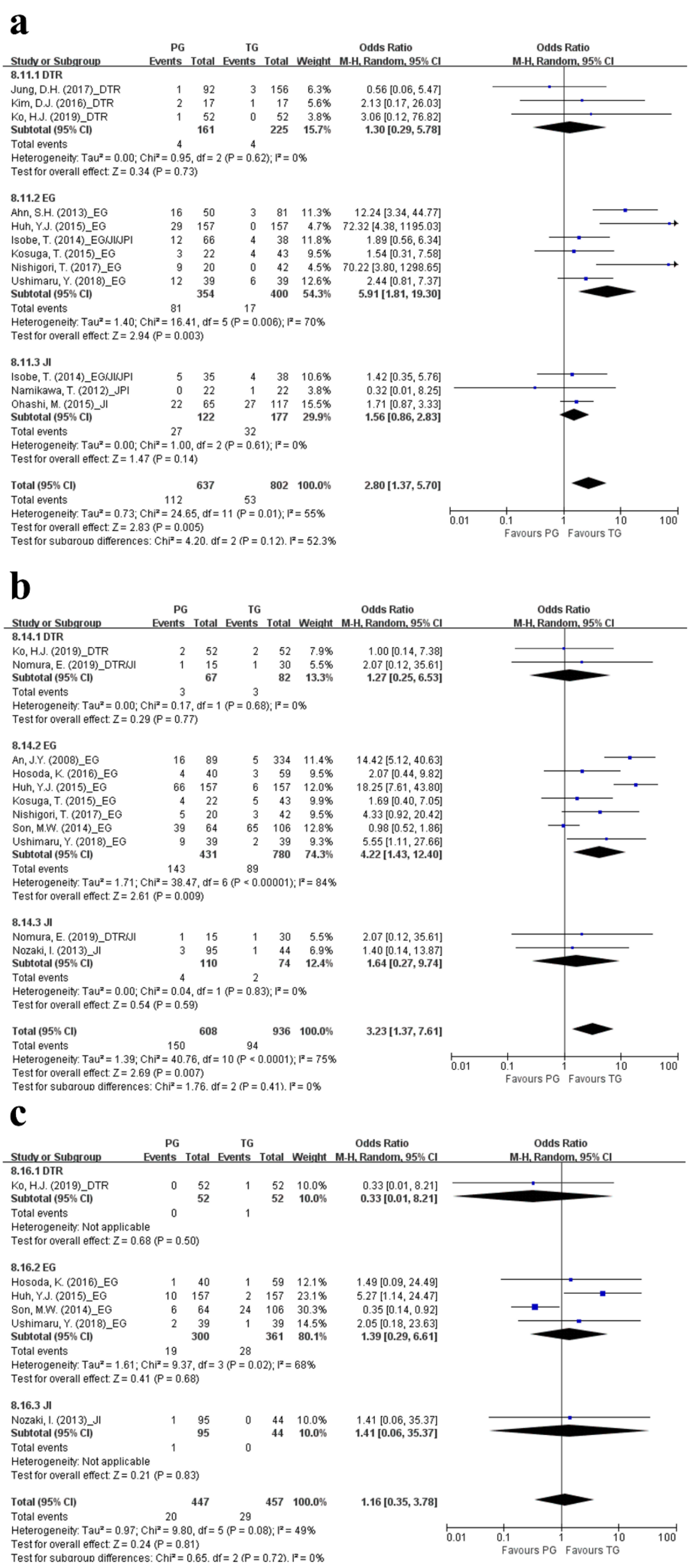

Supplementary Figure S4. Forest plots for comparing **a)** reflux symptom, **b)** reflux esophagitis, and **c)** severe reflux esophagitis using Los Angeles classification grade ≥ C between proximal gastrectomy and total gastrectomy. The meta-analysis was performed using the Mantel–Haenszel random-effects model. Odds ratios are shown with 95% confidence intervals. PG, proximal gastrectomy; TG, total gastrectomy; DTR, double-tract reconstruction; EG, esophagogastrostomy; JI, jejunal interposition.
